# Unique effect of aspirin on local 15‐oxo‐eicosatetraenoic acid synthesis in asthma patients with aspirin hypersensitivity

**DOI:** 10.1002/clt2.70004

**Published:** 2024-12-25

**Authors:** Piotr Szatkowski, Anna Gielicz, Adam Stępień, Patryk Hartwich, Radosław Kacorzyk, Hanna Plutecka, Adam Ćmiel, Gabriela Trąd‐Wójcik, Marek Sanak, Lucyna Mastalerz

**Affiliations:** ^1^ 2nd Department of Internal Medicine Jagiellonian University Medical College Krakow Poland; ^2^ Doctoral School of Medical and Health Sciences Jagiellonian University Krakow Poland; ^3^ Department of Otolaryngology Jagiellonian University Medical College Krakow Poland; ^4^ Department of Applied Mathematics AGH University of Science and Technology Krakow Poland

**Keywords:** 15‐oxo‐ETE, induced sputum, nonsteroidal anti‐inflammatory drug–exacerbated respiratory disease, oral aspirin challenge

## Abstract

**Background:**

Nonsteroidal anti‐inflammatory drugs–exacerbated respiratory disease (NSAIDs‐ERD) is characterized by altered arachidonic acid (AA) metabolism. Aspirin hypersensitivity is diagnosed using aspirin challenge, while induced sputum is collected to perform cell counts and to identify local biomarkers in induced sputum supernatant (ISS). This study aimed to assess the levels of a newly identified eicosanoid, 15‐oxo‐eicosatetraenoic acid (15‐oxo‐ETE), in ISS at baseline and during aspirin‐induced bronchospasm in patients with NSAIDs‐ERD.

**Methods:**

Oral aspirin challenge was performed in 27 patients with NSAIDs‐ERD and in 17 patients with aspirin‐tolerant asthma (ATA) serving as controls. Sputum was collected before and after aspirin challenge to determine eosinophil, neutrophil, macrophage, and lymphocyte counts as well as the concentration of AA metabolites via 15‐lipoxygenase‐1 (15‐LOX‐1) and 5‐LOX pathways in ISS. Chromatography–tandem mass spectrometry was used to measure ISS levels of 15‐oxo‐ETE, 15‐hydroxyeicosatetranoic acid (15‐HETE), and leukotriene E_4_ (LTE_4_).

**Results:**

At baseline, ISS levels of 15‐oxo‐ETE were higher in NSAIDs‐ERD than in ATA (*p* = 0.04). In contrast, baseline 15‐HETE levels in ISS were lower in patients with NSAIDs‐ERD (*p* = 0.03). After aspirin challenge, 15‐oxo‐ETE levels decreased only in patients with NSAIDs‐ERD (*p* = 0.001) who developed bronchospasm. In both study groups, there was a reduction in sputum macrophage count after aspirin challenge (*p* = 0.03 and *p* = 0.02, respectively) irrespective of bronchospasm.

**Conclusions:**

Patients with NSAIDs‐ERD are characterized by higher baseline 15‐oxo‐ETE levels in ISS than patients with ATA. Aspirin‐induced bronchospasm inhibited the local generation of 15‐oxo‐ETE.

## 1 INTRODUCTION

Nonsteroidal anti‐inflammatory drugs (NSAIDs)–exacerbated respiratory disease (NSAIDs‐ERD) is a chronic medical condition that consists of asthma, chronic rhinosinusitis with nasal polyps (CRSwNPs), and hypersensitivity reactions to the cyclooxygenase 1–inhibitory effects of NSAIDs.^1^, ^2^, ^3^ A typical component of NSAIDs‐ERD is difficult‐to‐treat CRSwNPs characterized by different inflammatory burden,^4^ and the presence of both eosinophilic and noneosinophilic bronchial inflammatory phenotype of asthma.^5^, ^6^, ^7^, ^8^

Although the pathogenesis of aspirin hypersensitivity is not fully understood, it is known that the syndrome is associated with type 2 (T2) inflammation of the airways. Interestingly, macrophages have a common altered phenotype resulting in a differential expression of T2 immune response cytokines, including interleukins IL‐4 and IL‐13, in patients with NSAIDs‐ERD.^9^ Importantly, numerous inflammatory cells of the upper and lower respiratory tract were reported to be involved in the pathogenesis of NSAIDs‐ERD, including eosinophils,^6^, ^8^ mast cells,^10^, ^11^, ^12^ innate lymphoid T2 cells,^13^, ^14^ platelets,^15^ epithelial cells,^16^ basophils,^17^ and interleukin‐5‐receptor‐α–positive plasma cells.^17^, ^18^

NSAIDs‐ERD is also associated with a complex dysregulation of the arachidonic acid (AA) metabolic pathways.^4^ There is an imbalance between the overproduction of proinflammatory eicosanoids in induced sputum supernatant (ISS), especially leukotriene LTE_4_ and prostaglandin PGD_2_, and anti‐inflammatory prostaglandin PGE_2_.^5^, ^19^ Abnormal levels of anti‐inflammatory regulators, including cyclooxygenase‐2 (COX‐2)–derived PGE_2_ and 15‐lipoxygenase (15‐LOX)–derived lipoxins were also documented.^20^, ^21^

**FIGURE 1:**
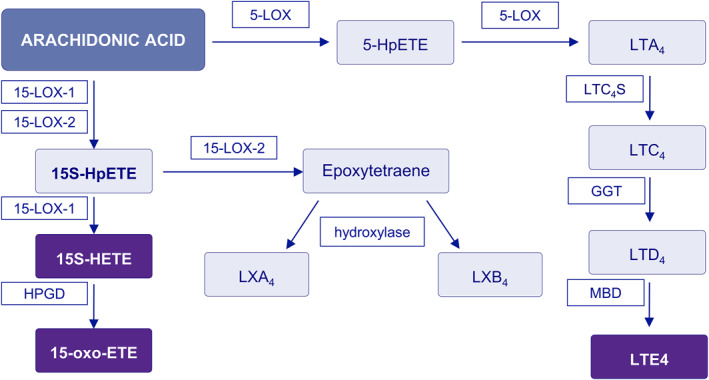
Arachidonic acid metabolism. 5‐LOX, 5‐lipoxygenase; 5‐HpETE, 5‐hydroperoxyeicosatetraenoic acid; 15S‐HETE, 15S‐hydroxyeicosatetraenoic acid; 15‐LOX‐1, 15‐lipoxygenase‐1; 15‐LOX‐2, 15‐lipoxygenase‐2; 15‐oxo‐ETE, 15‐oxo‐eicosatetraenoic acid; 15S‐HpETE, 15S‐hydroperoxyeicosatetraenoic acid; GGT, *γ* glutamyltransferase; HPGD, 15‐hydroxyprostaglandin dehydrogenase; LXA_4_, lipoxin A_4_; LXB_4_, lipoxin B_4_; LTC4S, leukotriene C4 synthase; MBD, membrane‐bound dipeptidase.

15‐LOX‐1 and 15‐LOX‐2 are encoded by the *ALOX1*5A and *ALOX1*5B genes, respectively. Those two enzyme isoforms have similar structural features and oxygenating polyunsaturated fatty acids.^22^ In this pathway, AA is converted by 15‐LOX‐1 and 15‐LOX‐2 into the (S)‐enantiomer of 15‐HPETE (15S‐HpETE), which is subsequently converted by 15‐LOX‐1 to 15‐HETE.^23^ Finally, 15‐HETE is converted to 15‐oxo‐eicosatetraenoic acid (15‐oxo‐ETE) by 15‐hydroxyprostaglandin dehydrogenase (HPGD).^23^, ^24^ 15‐LOX‐2 is responsible for the conversion of 15S‐HpETE to lipoxins (Figure 1).^25^, ^26^

Eosinophilia is characteristic of patients with NSAIDs‐ERD, and peripheral blood eosinophil count is higher in patients with NSAIDs‐ERD than in those with aspirin tolerant asthma (ATA).^8^, ^19^, ^27^ Notably, it was reported that AA in resting eosinophils is preferentially metabolized by 15‐LOX but is strongly shunted to 5‐LOX when calcium flux is present.^28^ Oxidation of AA by 5‐LOX leads to the formation of the unstable intermediate leukotriene LTA_4_. In neutrophils, LTA_4_ is preferentially hydrolyzed by LTA_4_ hydrolase to form LTB_4_, whereas in eosinophils, monocytes, basophils, and mast cells, it is conjugated to reduced glutathione by the terminal enzyme LTC_4_ synthase (LTC_4_S) and quickly converted to LTC_4_. At this point, LTC_4_ is exported from the cell and converted by *γ*‐glutamyltransferase to LTD_4_, which is finally converted by membrane‐bound dipeptidase to the stable end‐metabolite LTE_4_.^15^, ^29^, ^30^

Several studies have reported the overproduction of proinflammatory LTE_4_ in NSAIDs‐ERD compared with controls.^6^, ^15^, ^19^ Therefore, we decided to measure LTE_4_ levels in ISS. It was reported that the levels of a newly identified eicosanoid, 15‐oxo‐ETE, were markedly higher in the nasal polyp tissue of patients with NSAIDs‐ERD as compared with controls.^16^ Thus, we also aimed to assess 15‐oxo‐ETE levels as the end product of AA metabolite via 15‐LOX‐1 in ISS in patients with NSAIDs‐ERD. The levels of 15‐oxo‐ETE at baseline and during aspirin‐induced bronchospasm were compared. In addition, we investigated differences in 15‐oxo‐ETE levels between patients with NSAIDs‐ERD and those with ATA.

## 2 METHODS

### 2.1 Study groups

The study group included 27 patients with NSAIDs‐ERD, and the control group included 17 patients with ATA. Asthma was defined according to GINA 2022 (https://ginasthma.org/gina‐reports)^31^ and CRSwNP was recognize based on European Position Paper on Rhinosinusitis and Nasal Polyps 2020 (https://doi.org/10.4193/Rhin20.600).^32^ Exclusion criteria were: liver and kidney failure, diabetes, neoplastic diseases, autoimmune diseases, cystic fibrosis, pulmonary chronic disease, infectious diseases, pregnancy and lactation, addiction to drugs, alcohol abuse, tobacco smoking and/or a difficulty to face study protocol requirements. All patients had stable asthma. None of the study participants did not present acute symptoms of infections and exacerbation in 6‐week run‐in period before aspirin challenge. None of the participants of the study did not smoke tobacco. All participants completed the Asthma Control Test (ACT), 7‐item Asthma Control Questionnaire (ACQ‐7), and Mini Asthma Quality of Life Questionnaire (Mini‐AQLQ). The severity of asthma was assessed according to the 2022 Global Initiative for Asthma guidelines (GINA),^31^ and the severity of CRSwNPs was assessed using the 22‐item Sino‐Nasal Outcome Test (SNOT‐22). Computed tomography scans of the sinuses were assessed according to the Lund‐Mackay score by two experienced radiologists.^33^ To recognize aspirin hypersensitivity, we used the clinical “gold standard” test, which is an oral aspirin challenge. We administered five exponentially increasing doses of aspirin (27, 44, 117, 312, 500 mg) every 1.5–2 h until a cumulative dose achieved value 1000 mg. The challenge was considered positive if there was a reduction in baseline FEV1 by at least 20%. A provocative dose of aspirin causing a 20% reduction in baseline FEV1 (PD20) was calculated. The results were considered negative when the cumulative dose of aspirin (1000 mg) was achieved and there was no significant reduction in baseline FEV1 and no clinical symptoms were observed.^34^ Based on that, when patients achieved a positive provocation test, we classified them as these with aspirin intolerance, opposite to negative test, as these with aspirin tolerance.

All patients were treated with nasal and inhaled corticosteroids as well as long‐acting *β*_2_‐agonists for a 6‐week run‐in period before aspirin challenge. Two patients with NSAIDs‐ERD were treated with oral corticosteroids at a dose of 4 mg methylprednisolone per day. None of the patients with NSAIDs‐ERD or ATA used antileukotrienes or biological drugs during the 6 months preceding sputum collection. Moreover, none of the patients with ATA used aspirin or other NSAIDs in the 6 weeks preceding the study. On the day of the aspirin challenge, all participants had a forced expiratory volume in the first second (FEV_1_) of 70% or higher. The characteristics of the study groups are presented in Table 1.

**TABLE 1:** Demographics, clinical, and biochemical characteristics of the study groups.

Variable		NSAIDs‐ERD (*n* = 27)	ATA (*n* = 17)	*p‐*Value*
Age, years		47 (39–56)	46 (42–57)	0.95
Sex, *n* (%)	Female	24 (88.9)	12 (70.6)	0.23
	Male	3 (11.1)	5 (29.4)	
BMI, kg/m^2^		25.8 (22.7–29.4)	26.7 (24.8–28.9)	0.99
Severity of asthma, *n* (%)	Mild	0	3 (17.6)	0.08
	Moderate	13 (48.2)	8 (47.1)	
	Severe	14 (51.8)	6 (35.3)	
Age of asthma onset, years		33 (21–43)	37 (29–42)	0.69
Asthma duration, years		14 (9–24)	10 (5–21)	0.22
ICS dose (fluticasone propionate or equivalent), μg		600 (400–1250)	400.0 (250–800)	0.016
Baseline FEV_1_, %		96 (86–105)	96 (87–106)	0.59
History of sinonasal surgery (yes/no), *n* (%)		26/1 (96)	13/4 (76)	0.065
PD20, mg		350 (150–420)	‐	‐
Immunoglobulin E at baseline, IU/mL		107 (56–150)	43 (21–122)	0.13
Blood eosinophils at baseline, %		5.7 (3.4–9.0)	3.2 (2.1–5.2)	0.02
Positive skin prick test, *n* (%)		9 (33)	8 (47)	0.36
Mini‐AQLQ score		5.2 (4.2–6.3)	5.3 (3.7–6.2)	0.73
ACQ‐7 score		0.57 (0.43–1.70)	0.86 (0.14–1.70)	0.72
ACT score		23 (20–25)	24 (22–25)	0.23
SNOT‐22 score		49 (36–72)	39 (29–46)	0.04
Lund‐Mackay score		13 (12–19.5)	8 (2–14)	0.01

*Note*: Data are presented as median and quartile 1, quartile 3 (Q1, Q3) unless indicated otherwise.

Abbreviations: ACQ‐7, 7‐item Asthma Control Questionnaire; ACT, Asthma Control Test; ATA, aspirin‐tolerant asthma; BMI, body mass index; FEV_1_, forced expiratory volume in the first second; ICS, inhaled corticosteroids; Mini‐AQLQ, Mini Quality of Life Asthma Questionnaire; NSAIDs‐ERD, nonsteroidal anti‐inflammatory drug–exacerbated respiratory disease; PD20, provocative dose causing a 20% fall in baseline FEV_1_; SNOT‐22, 22‐item Sino‐Nasal Outcome Test.

*p < 0.05 was considered significant.

The study that provided data used in the current analysis was approved by the Jagiellonian University Ethics Committee (Nr 1072.6120.48.2021). All participants provided written informed consent to take part in the study, and the study protocol was in line with the Declaration of Helsinki.

### 2.2 Study design

All patients with NSAIDs‐ERD or ATA underwent a single‐blind, placebo‐controlled oral aspirin challenge test over two consecutive days.^34^ The challenge was considered positive if there was a reduction in baseline FEV_1_ by at least 20%. A provocative dose (PD20) of aspirin causing a 20% reduction in baseline FEV_1_ was calculated. The results were considered negative when the cumulative dose of aspirin (1000 mg) was achieved and there was no significant reduction in baseline FEV_1_ and no clinical symptoms were observed.

Induced sputum and urine were collected at baseline and then after aspirin challenge when symptoms of aspirin hypersensitivity occurred (in patients with NSAIDs‐ERD) or when no symptoms occurred and the cumulative aspirin dose of 1000 mg was achieved (in patients with ATA).

### 2.3 Induced sputum collection and analysis

Induced sputum was collected according to the methodology recommended by the European Respiratory Society^35^ and used in our previous research.^5^, ^6^, ^7^, ^19^, ^36^ Sputum samples were processed to obtain cytospin slides for a differential cell count (macrophage, lymphocyte, eosinophil, and neutrophil) and a supernatant for the evaluation of 15‐HETE, 15‐oxo‐ETE and LTE_4_.

ISS samples for 15‐oxo‐ETE as well as 15‐HETE and LTE_4_ assessment were aliquoted and stored at −80°C until analysis. ISS corresponded to the mucous plug diluted at a 1:10 weight/volume ratio.^19^, ^37^ SPE C18 columns (SEP‐PAK PLUS C18; Waters) were used for extraction. The SPE columns were washed with 1‐mL methanol and 1‐mL MilliQ water. Next, samples with an internal standard mixture were loaded into the cartridges and washed with MilliQ water. The aqueous plugs were pulled from the SPE cartridges under a gentle stream of nitrogen for 15 min. Subsequently, the washing phase was conducted using a mixture of methanol and tert‐butyl methyl ether (v/v, 4:1) added to Eppendorf tubes. Collected samples were dissolved in 50‐μL methanol and assessed using high‐performance liquid chromatography–tandem mass spectrometry (Triple Quad 5500+; AB Sciex, Washington, DC, United States) with an electrospray ion source. The analytical method was described in detail elsewhere.^5^, ^19^ 15‐HETE, 15‐oxo‐ETE, and LTE_4_ levels were calculated from the area under the peak using a stable isotope dilution method. The total protein concentration in ISS was measured using a Cobas Integra 400plus biochemical analyzer (Roche, Basel, Switzerland) and expressed in milligrams per milliliter. Instruments were set to multiple‐reaction monitoring modes and negative ionization. Gradient elution consisted of two mobile phases: (A) acetonitrile/water/acetic acid (20/80/0.0001); and (B) acetonitrile/iso‐propanol/acetic acid (55/45/0.0001, v/v) under a constant flow rate of 0.11 mL/min. The mobile phase binary linear gradient was 1 min 8% B, 9.5 min 8%–95% B, 0.5 min 95% B, 0.5 min 95%–100% B, and 2 min 100% B. The ISS levels of 15‐HETE, 15‐oxo‐ETE and LTE_4_ were expressed as picograms per milligram of protein. The cell counts were expressed as a percentage of 800 cells.

### 2.4 Urine samples collecting and analysis

Urine samples were collected before and after the aspirin provocation test and stored at −80°C until analysis. The samples were processed for the evaluation of 15‐HETE, 15‐oxo‐ETE and LTE_4_ measured by high performance liquid chromatography‐tandem mass spectrometry (HPLC‐MS/MS) (AB SCIEX, QTrap 4000).^38^

Briefly, the urine samples with internal standard mixture were extracted with the mixture of tert‐Butyl methyl eter:methanol (v/v, 4:1) as liquid–liquid extraction. The organic phase was transferred to a fresh tube, evaporated in nitrogen stream and dissolved in 40 μL of methanol. Urinary 15‐oxo‐ETE, 15‐HETE and LTE_4_ levels were calculated from the area under the peak of chromatograms using 5‐point calibration curves. Creatinine levels were measured using an Integra 400+ Chemistry Analyzer (Roche Diagnostics, Bazel, Switzerland). The results were recalculated in picograms per mg of creatinine (pg/mg creatinine). All HPLC‐MS/MS settings and calculation method are presented above.

### 2.5 Study design AA‐HETEs eicosanoids

Induced sputum and urine were collected twice, at baseline and after the challenge when symptoms of aspirin hypersensitivity developed or if no symptoms occurred in the ATA group. Induced sputum supernatant (ISS) and urine for AA‐HETEs eicosanoids (15‐HETE, 15‐oxo‐ETE, LTE_4_) concentration were carried out in both study groups.

### 2.6 Statistical analysis

Descriptive statistics for demographic, clinical, and laboratory characteristics were presented as medians with 25th and 75th percentiles for continuous variables and frequencies with percentages for categorical variables. The general linear model was used for group comparisons. Logarithmic or Box–Cox transformations were used as variance‐stabilizing transformations when needed. When the transformations were unsuccessful, the nonparametric Kruskal‐Wallis analysis of variance was used. The post hoc Tukey test or the nonparametric Kruskal‐Wallis test was used to control the family wise error rate for multiple comparisons. Correlations between variables were assessed with the Spearman rank order correlation coefficient. Benjamini–Hochberg adjusted *p*‐values were used to control the false discovery rate in the search for significant correlations. A *p*‐value of 0.05 or lower was considered significant. Logit modeling (logarithmic of odds) were procced for notice potential predictors to recognize patients with NSAIDs‐ERD. Akaike criteria were used to choose the best logit modeling. Box‐Cox transformations were used in the analysis of influence between variables and Mallows criteria were used to build a linear model with an optimal model selection procedure to avoid the catalyst effect. Statistical analysis was performed with Statistica 13.3 (TIBCO Software Inc.).

## 3 RESULTS

### 3.1 Clinical characteristics of study participants

The demographic, clinical, and biochemical characteristics of the NSAIDs‐ERD and ATA groups are presented in Table 1. There were no significant differences in age, sex, and body mass index ,BMI between the groups. Moreover, no significant differences were noted in the ACT, ACQ‐7, and Mini‐AQLQ scores. The dose of inhaled corticosteroids was significantly higher in patients with NSAIDs‐ERD than in those with ATA. There was a trend toward greater asthma severity in patients with NSAIDs‐ERD, but the differences were not significant. Patients with NSAIDs‐ERD also had a significantly higher blood eosinophil counts than those with ATA. Significant differences were noted in SNOT‐22 and Lund‐Mackay scores, with higher values in NSAIDs‐ERD versus ATA. Two patients with oral glicocorticosteroids from the NSAIDs‐ERD group did not change the results. We verified that by statistical analysis with exclusion of these two patients, but the results were unchanged. Therefore, we decided to include them in the presented data.

### 3.2 15‐HETE in induced sputum supernatant

**FIGURE 2:**
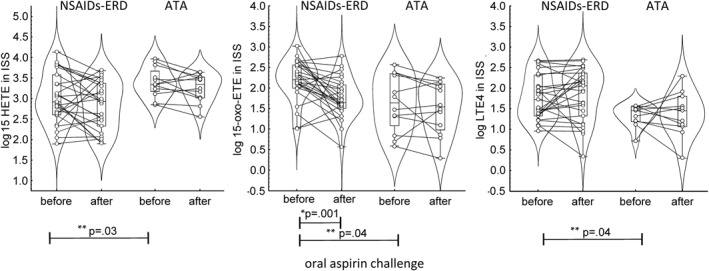
Sputum 15‐HETE, 15‐oxo‐ETE, LTE_4_ levels before and after oral aspirin challenge in patients with NSAIDs‐ERD and ATA. *differences before and after oral aspirin challenge in NSAIDs‐ERD **differences between NSAIDs‐ERD versus ATA at baseline. 15‐oxo‐ETE, 15‐oxo‐eicosatetraenoic acid; 15‐HETE, 15‐hydroxyeicosatetraenoic acid; ATA, aspirin‐tolerant asthma; LTE4, leukotriene E4; ISS, induced sputum supernatant; NSAIDs‐ERD, nonsteroidal anti‐inflammatory drug–exacerbated respiratory disease.

At baseline, 15‐HETE levels in the NSAIDs‐ERD group were lower compared with those in ATA (*p* = 0.03). However, there were no significant differences between 15‐HETE levels in ISS before and after aspirin challenge in both study groups (Figure 2). Data are presented in Table 2.

**TABLE 2:** Differences in 15‐hydroxyeicosatetraenoic acid (15‐HETE), 15‐oxo‐eicosatetraenoic acid (15‐oxo‐ETE), leukotriene E_4_ (LTE_4_) levels in ISS and in sputum cell counts before and after aspirin challenge.

	NSAIDs‐ERD (*n* = 27)			ATA (*n* = 17)			*p*‐Value*
	Before	After	*p*‐Value	Before	After	*p*‐Value	
15‐HETE, pg/mg protein							
Median (25%–75%)	829.6 (400.0–3772.8)	847.5 (214.4–2332.4)	0.09	2189.1 (1528.5–3225.5)	1830.4 (1042.4–3180.1)	0.19	0.03
15‐oxo‐ETE, pg/mg protein							
Median (25%–75%)	158.80 (99.15–362.2)	44.16 (31.88–118.62)	0.001	80.0 (21.6–204.7)	88.7 (15.9–142.8)	0.12	0.04
LTE_4_, pg/mg protein							
Median (25%–75%)	52.8 (21.0–206.6)	113.4 (21.1–231.0)	0.15	28.5 (15.3–34.9)	28.3 (11.7–63.8)	0.66	0.04
Sputum cells, (%; percentage of 800 cells)							
Macrophages	50.8 (36.3–59.1)	44.6 (29.8–53.3)	0.03	31.6 (27.1–61.2)	19.7 (8.7–35.4)	0.02	0.38
Neutrophils	21.5 (11.5–39.4)	25.7 (11.9–43.0)	0.19	50.8 (30.6–59.3)	56.1 (51.3–79.0)	0.12	0.001
Eosinophils	0.87 (0.0–6.9)	0.62 (0.0–7.5)	0.35	1.41 (0.9–3.9)	2.8 (1.7–6.6)	0.70	0.49
Lymphocytes	0.75 (0.5–1.1)	0.5 (0.4–1.0)	0.13	1.4 (0.6–3.2)	1.8 (1.0–2.7)	0.10	0.016

*Note*: Data are presented as median and quartile 1, quartile 3 (Q1, Q3) for arachidonic acid– hydroxyeicosatetraenoic acid (AA‐HETE) eicosanoids and mean ± SD for sputum cells. * differences between groups at baseline.

Abbreviations: ATA, aspirin‐tolerant asthma; NSAIDs‐ERD, nonsteroidal anti‐inflammatory drug–exacerbated respiratory disease.

### 3.3 15‐oxo‐ETE levels in induced sputum supernatant

At baseline, 15‐oxo‐ETE levels were higher in patients with NSAIDs‐ERD than in those with ATA (*p* = 0.04) (Table 2). There was a significant reduction in 15‐oxo‐ETE levels after aspirin challenge versus baseline only in patients with NSAIDs‐ERD (*p* = 0.001) who developed bronchospasm but not in those with ATA (Figure 2).

### 3.4 LTE_4_ levels in induced sputum supernatant

At baseline, LTE_4_ levels were higher in NSAIDs‐ERD than in ATA (*p* = 0.04) (Table 2). There were no significant differences between LTE_4_ levels in ISS before and after aspirin challenge in both study groups (Figure 2).

### 3.5 AA‐metabolites in urine

The level of urinary 15‐HETE remained unchanged during aspirin‐induced bronchospasm (median 15.0 pg/mg creatinine, before; 19.4 pg/mg creatinine, after; *p* = 0.42).

In the NSAIDs‐ERD group, before the aspirin provocation test, the level of 15‐oxo‐ETE in urine was 6.2 pg/mg creatinine (median) and after 10.7 pg/mg creatinine (median), differences were not significant statistically (*p* = 0.69).

**FIGURE 3:**
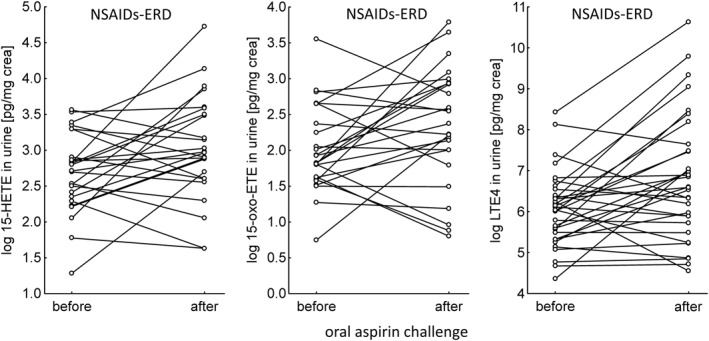
Urinary 15‐HETE, 15‐oxo‐ETE, LTE_4_ levels before and after oral aspirin challenge in patients with NSAIDs‐ERD. 15‐oxo‐ETE, 15‐oxo‐eicosatetraenoic acid; 15‐HETE, 15‐hydroxyeicosatetraenoic acid; ISS, induced sputum supernatant; LTE_4_, leukotriene E_4_; NSAIDs‐ERD, nonsteroidal anti‐inflammatory drug–exacerbated respiratory disease.

Urinary LTE_4_ during the aspirin provocation test increased in the NSAIDs‐ERD group and differences were statistically significant (median 407.2 pg/mg creatinine, before; 942.5 pg/mg creatinine after; *p* = 0.007), see Figure 3. Explanations about results in ATA, see limitations.

### 3.6 Sputum cell counts at baseline and after oral aspirin challenge

**FIGURE 4:**
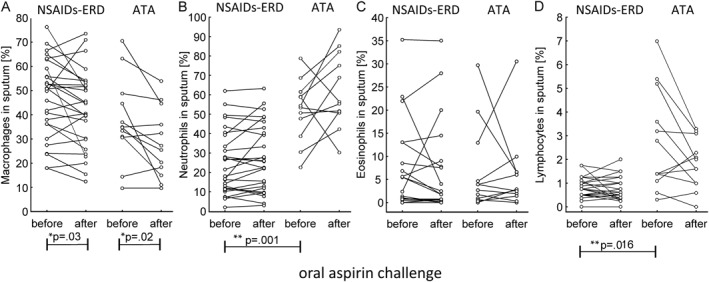
Sputum cell counts (A, macrophages; B, neutrophils; C, eosinophils; D, lymphocytes) at baseline and after oral aspirin provocation test in patients with NSAIDs‐ERD and ATA; * differences before and after aspirin challenge in NSAIDs‐ERD or ATA. **differences between NSAIDs‐ERD versus ATA at baseline. ATA, aspirin‐tolerant asthma; NSAIDs‐ERD, Nonsteroidal anti‐inflammatory drug–exacerbated respiratory disease.

At baseline, patients with NSAIDs‐ERD showed lower neutrophil (*p* = 0.001) and lymphocyte (*p* = 0.016) counts compared with the ATA group. After aspirin challenge, sputum cytology revealed a significant reduction only in macrophage count in both the NSAIDs‐ERD (*p* = 0.03) and ATA (*p* = 0.02) groups (Figure 4). There were no significant differences in neutrophil, lymphocyte, or eosinophil counts during aspirin challenge in any of the study groups.

### 3.7 Correlations in the study groups

There were no significant correlations between baseline 15‐oxo‐ETE, 15‐HETE, LTE_4_ levels in ISS and the demographic (age, sex, BMI), clinical (age of asthma onset, asthma duration, Mini‐AQLQ score, ACQ‐7 score, ACT score, asthma severity based on GINA 2022, inhaled corticosteroid dose, skin prick test results, baseline FEV_1_, CRSwNP, number of sinonasal surgeries), biochemical (total IgE in serum), blood eosinophils, and sputum cytology (neutrophils, lymphocytes, basophils, and macrophages) characteristics of patients in any of the study groups.

In the NSAIDs‐ERD group, 15‐HETE levels correlated with sputum eosinophil count at baseline (*r* = 0.67, *p* = 0.001). No other significant correlations were found between 15‐HETE levels in ISS and sputum cell counts before and after oral aspirin challenge. In addition, no correlation was found between 15‐oxo‐ETE levels in ISS and sputum cell counts before and during aspirin‐induced bronchospasm in the NSAIDs‐ERD group.

At baseline, there was no correlation between 15‐HETE and 15‐oxo‐ETE levels in ISS in the NSAIDs‐ERD group, but a significant positive correlation between these parameters was noted after aspirin challenge (*r* = 0.71, *p* = 0.002). No significant correlations were found between 15‐oxo‐ETE and PD_20_ of aspirin (*r* = 0.03, *p* = 0.88), and between 15‐oxo‐ETE and the time of symptom onset (*r* = −0.29, *p* = 0.14) in patients with NSAIDs‐ERD.

In the NSAIDs‐ERD group, there was a significant positive correlation between LTE_4_ and 15‐HETE levels in ISS before (*r* = 0.70, *p* = 0.001) and after (*r* = 0.51, *p* = 0.04) aspirin challenge. Moreover, LTE_4_ levels in ISS correlated positively with sputum eosinophil count (*r* = 0.73, *p* = 0.001) at baseline.

In the ATA group, there was a significant positive correlation between baseline LTE_4_ and 15‐HETE (*r* = 0.71, *p* = 0.002) levels in ISS.

Numbers of exacerbations in the last 12 months did not influence the level of 15‐HETE, 15‐ox‐ETE, LTE_4_ in ISS. Moreover, there were no marked correlations between the number of exacerbations and sputum cells.

### 3.8 Predictors for NSAIDs‐ERD

Only 15‐oxo‐ETE in ISS is a good predictor to classify patients into the NSAIDs‐ERD group (*p* < 0.001), variable like dose of ICS, SNOT‐22, and L‐M score separately as predictors for NSAIDs‐ERD were insignificant. Constellation of 15‐oxo‐ETE in ISS with dose of ICS, L‐M score or SNOT‐22 the odds to recognize patients into NSAIDs‐ERD group decreased to insignificant level and these variables together could not be used as a predictors to recognize NSAIDs‐ERD. Other variables such as group (NSAIDs‐ERD/ATA), age, sex, BMI, severity and asthma control, total IgE in serum, blood eosinophils, scores: ACT, miniAQLQ did not influence 15‐oxo‐ETE in ISS.

Level of 15‐oxo‐ETE in ISS was an independent variable, without any marked influence by age, sex, severity, asthma control, FEV1%, total IgE, blood eosinophils, dose of ICS, scores: ACT, mini‐AQLQ, SNOT‐22, and L‐M score and it was the best predictor to categorize patients into the NSAIDs‐ERD group.

## 4 DISCUSSION

**FIGURE 5:**
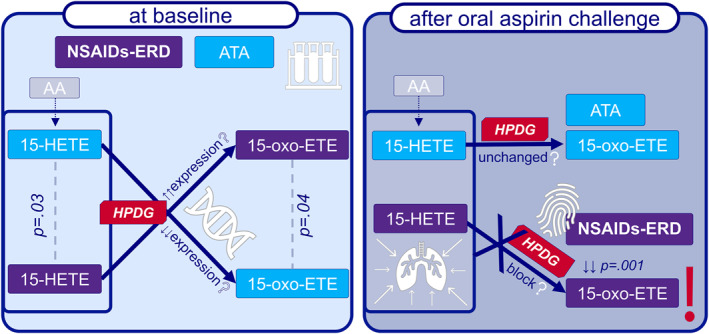
Results of the study with speculated reason. We noticed a lower level of 15‐HETE and higher level of 15‐oxo‐ETE at baseline in NSAIDs‐ERD compared to ATA in ISS with local metabolic block during bronchospasm in synthesis of 15‐oxo‐ETE from 15‐HETE only in NSAIDs‐ERD. Those differences could be common with different expressions of *HPDG*. 15‐oxo‐ETE, 15‐oxo‐eicosatetraenoic acid; 15‐HETE, 15‐hydroxyeicosatetraenoic acid; AA, arachidonic acid; ATA, aspirin‐tolerant asthma; HPDG, 15‐hydroxyprostaglandin dehydrogenase; NSAIDs‐ERD, nonsteroidal anti‐inflammatory drug–exacerbated respiratory disease.

To our knowledge, this is the first report to assess local sputum 15‐oxo‐ETE levels at baseline and following oral aspirin challenge in patients with NSAIDs‐ERD. At baseline, local 15‐oxo‐ETE levels in ISS were significantly higher in patients with NSAIDs‐ERD than in the control group. An unexpected finding in the NSAIDs‐ERD group was a marked reduction in 15‐oxo‐ETE levels in ISS during aspirin‐induced bronchospasm. We hypothesize that this phenomenon could be related to aspirin‐induced changes in the expression of genes that synthesize products involved in AA metabolism, especially *ALOX‐*15A/B and *HPDG*. Our hypothesis is presented in Figure 5.

15‐oxo‐ETE is a proinflammatory eicosanoid^39^ that exhibits paradoxical behavior after aspirin challenge when symptoms of aspirin hypersensitivity occur in patients with NSAIDs‐ERD. During aspirin‐induced bronchospasm in patients with NSAIDs‐ERD, 15‐oxo‐ETE showed similar effects to the anti‐inflammatory PGE_2_ in ISS,^5^, ^19^ although they both originate from different AA metabolic pathways: 15‐oxo‐ETE from 15‐LOX‐1 and PGE_2_ from COX‐2. It is important to note that there is a link between 15‐oxo‐ETE and PGE_2_. In human neutrophils, *ALOX‐15* expression can be induced by anti‐inflammatory PGE_2_ via cAMP elevation.^40^ The induction of *ALOX‐15* in neutrophils may contribute to the redirection of the 15‐LOX pathway from 15‐LOX‐1 to 15‐LOX‐2, which promotes the synthesis of anti‐inflammatory lipoxins.^40^ It was reported that 15‐LOX‐1‐induced inhibition profiles in eosinophils and neutrophils are not identical.^41^ It would be interesting to examine this phenomenon in patients with NSAIDs‐ERD, especially since neutrophil counts in ISS at baseline were lower in asthma patients with aspirin hypersensitivity. Neutrophils^42^ may also be involved in the pathogenesis of aspirin hypersensitivity in addition to other cells.

A reduction in sputum 15‐oxo‐ETE levels after aspirin challenge may indicate that other pro‐inflammatory eicosanoids, rather than 15‐oxo‐ETE, are responsible for acute aspirin‐induced bronchospasm in patients with NSAIDs‐ERD. In our previous research, we documented an increase in the levels of proinflammatory cysteinyl leukotrienes with a simultaneous reduction in anti‐inflammatory PGE_2_ levels in ISS during bronchospasm induced by oral aspirin challenge.^5^, ^19^ Moreover, we found that AA‐related genes, except *LTC*_*4*_ *S* and *ALOX15*, were grouped together, indicating the ability of aspirin to regulate the expression of these genes in NSAIDs‐ERD.^43^ However, in the present study, no significant correlation was found between the provocative dose of aspirin and the time of bronchospasm onset and sputum 15‐oxo‐ETE levels. On the other hand, it seems that high‐dose long‐term aspirin therapy following aspirin desensitization may change the pathway of AA metabolism via 15‐LOX and can lead to higher plasma 15‐HETE levels.^44^ Patients with NSAIDs‐ERD who responded to aspirin therapy were characterized by a high expression of the *HPGD* gene in sputum cells at baseline,^36^ and the *HPGD* gene encodes HPGD—the terminal enzyme for 15‐oxo‐ETE synthesis. In this study, we found a positive correlation between sputum 15‐HETE and 15‐oxo‐ETE levels during aspirin‐induced bronchospasm, which indirectly indicates that aspirin affects the 15‐LOX pathway of AA metabolism in patients with NSAIDs‐ERD.

Recently, it was hypothesized that epithelial and mast cell interactions lead to 15‐oxo‐ETE synthesis in the polyp tissue in patients with NSAIDs‐ERD. Indeed, the expression of the *ALOX15* gene encoding 15‐LOX was significantly elevated in the nasal polyp tissue of patients with NSAIDs‐ERD compared with controls. *ALOX15* was predominantly expressed by epithelial cells. Its expression was significantly correlated with lower FEV_1_ and radiographic severity of sinus disease in NSAIDs‐ERD.^16^ Notably, HPGD was predominantly expressed in mast cells and localized near the 15‐LOX epithelium in the nasal polyp tissue of these patients.^12^, ^16^

In our study, aspirin challenge resulted in a reduction in sputum macrophage count independent of aspirin hypersensitivity. Human macrophages express two AA 15‐LOX enzymes: 15‐LOX1 and 15‐LOX‐2. In macrophages, 15‐LOX‐1 is responsible for the synthesis of lipids necessary to produce specialized pro‐resolving mediators that facilitate the resolution of inflammation. On the other hand, 15‐LOX‐2 shows constitutive expression on macrophages, and the biological functions of this enzyme are not fully understood.^45^ In our study, after oral aspirin challenge, macrophages were not recruited into the respiratory tract (sputum) and remained in the lungs. It is possible that aspirin leads to a loss of the chemotactic gradient that drives macrophages to the sputum and leads to the functional reprogramming of myeloid progenitors in the bone marrow.^46^ The functional reprogramming phenomenon was reported to possibly contribute to chronic T2 airway inflammation in NSAIDs‐ERD.^46^, ^47^ Further studies are needed to assess changes in *ALOX15* expression and aspirin‐induced shift between LOX‐1 and LOX‐2 enzymes in sputum cells, especially in macrophage,^48^ in patients with NSAIDs‐ERD. Both local eicosanoid synthesis and sputum inflammatory cells are important for understanding the mechanism of aspirin‐induced bronchospasm. The abnormal eicosanoid profile in ISS results from the mutual interactions between eicosanoid‐generating cells.^5^, ^37^

At baseline, bronchial 15‐HETE concentrations in patients with NSAIDs‐ERD were significantly lower compared with those in ATA. Recently, it was reported that low 15‐HETE levels at baseline predict greater accumulation of group 2 innate lymphoid cells in the airways of patients with NSAIDs‐ERD who receive COX‐1 inhibitors.^14^ In the current study, sputum total lymphocyte counts did not change during aspirin‐induced bronchospasm; however, the immune‐phenotypes and cytokine profiles of these lymphocytes are unknown at this stage of the study.

Notably, correlations were found between sputum cells and 15‐oxo‐ETE levels in ISS. A positive correlation was only found between 15‐HETE levels in ISS and sputum eosinophil count at baseline. This correlation may indicate the main cell source of 15‐HETE. Moreover, sputum 15‐HETE levels did not change during aspirin‐induced bronchospasm compared with baseline and were positively correlated with markedly reduced 15‐oxo‐ETE levels in patients with NSAIDs‐ERD. This might confirm that aspirin exerts a unique effect on the expression of the *HPGD* gene.

We also found that proinflammatory LTE_4_ levels in ISS were significantly higher in NSAIDs‐ERD than in ATA and were positively correlated with sputum eosinophil count. These findings are in line with previous research in a similar population of patients.^6^, ^19^ Moreover, urinary LTE_4_ in the NSAIDs‐ERD group was markedly increased during aspirin‐induced bronchospasm, by other team.^49^ Previously, we reported that a higher level of LTE^4^ in urine is typical for patients with NSAIDs‐ERD compared to ATA^50^ and that an increased level of urinary LTE_4_ in NSAIDs‐ERD is common with aspirin desensitization.^36^ Thus, the resulting study in this project remains in connection with previously data.

Our results open the door for further research on AA metabolism via the 15‐LOX pathway in patients with NSAIDs‐ERD. Although it is known that the bronchial biosynthesis of 15‐oxo‐ETE is inhibited by aspirin, more questions than answers remain and the mechanism underlying aspirin‐induced bronchospasm is still poorly understood. Lipids associated with the distinct pathways of AA metabolism, including lipoxygenases, can be assessed and correlated with systemic (plasma, urine) and local (tissue, nasal lavage, ISS) generation of these metabolites simultaneously with different inflammatory cells. Indeed, cellular interactions through eicosanoid signaling via specific receptors contribute to the characteristic features of NSAIDs‐ERD. In the future, it is important to fully elucidate the receptor mechanism of action of 15‐oxo‐ETE in patients with NSAIDS‐ERD.

Our study has several limitations. Differences in the cumulative dose of aspirin in asthma patients with positive and negative results of the aspirin challenge^34^ can influence the final outcome. In patients with NSAIDs‐ERD, aspirin causes an episode of bronchoconstriction, which logically cannot occur in patients with ATA. Bronchospasm itself is a traumatic event for the airways, and it leads to inflammatory and even cellular changes. Therefore, when assessing sputum from patients with NSAIDs‐ERD, it is not possible to differentiate whether some of the changes are due to COX‐1 inhibition by aspirin or to trauma represented by bronchospasm. During the project, we collected urine samples in the NSAIDs‐ERD and ATA groups. Unfortunately, samples with urine from ATA patients were stored in a freezer which suffered from a technical failure.

In conclusion, an independent and novel AA‐metabolite, 15‐oxo‐ETE, a downstream product of the 15‐LOX‐1 pathway, can be detected locally in patients with asthma. The study revealed the over synthesis of sputum 15‐oxo‐ETE in patients with NSAIDs‐ERD compared with those with ATA. Aspirin‐induced bronchospasm is characterized by a reduction in the local neo‐synthesis of 15‐oxo‐ETE in NSAIDs‐ERD.

## AUTHOR CONTRIBUTIONS

**Piotr Szatkowski**: Formal analysis; methodology; validation; writing—original draft; software; visualization; data curation; investigation. **Anna Gielicz**: Methodology; validation; software; formal analysis; writing—original draft; investigation. **Adam Stępień**: Writing—original draft; formal analysis. **Patryk Hartwich**: Writing—original draft; formal analysis. **Radosław Kacorzyk**: Writing—review and editing; formal analysis; data curation; methodology; validation; software. **Hanna Plutecka**: Writing—original draft; methodology; validation; formal analysis; software; investigation. **Adam Ćmiel**: Writing—original draft; formal analysis; software; data curation; visualization. **Gabriela Trąd‐Wójcik**: Methodology; validation; visualization; writing—review and editing; Software; Formal analysis. **Marek Sanak**: Writing—original draft; Methodology; Validation; Formal analysis; software; investigation; visualization. **Lucyna Mastalerz**: Writing—original draft; conceptualization; investigation; supervision; methodology; validation; visualization; formal analysis; project administration; software; data curation; funding acquisition.

## CONFLICT OF INTEREST STATEMENT

None declared.

## Data Availability

The data that support the findings of this study are available from the corresponding author upon reasonable request.
